# The acrylamide content of smokeless tobacco products

**DOI:** 10.1186/s13065-015-0132-1

**Published:** 2015-10-12

**Authors:** Kevin McAdam, Harriet Kimpton, Carl Vas, David Rushforth, Andrew Porter, Brad Rodu

**Affiliations:** Group Research and Development, British American Tobacco, Regents Park Road, Southampton, SO15 8TL UK; 3810 St. Antoine W., Montreal, QC H4C 1B4 Canada; Department of Medicine, School of Medicine, University of Louisville, 505 South Hancock Street, Louisville, KY 40202 USA

**Keywords:** Acrylamide, Smokeless tobacco products, Snus, Snuff

## Abstract

**Background:**

There is considerable interest from a regulatory and public health perspective in harmful and potentially harmful constituents in tobacco products, including smokeless tobacco products (STPs). A wide range of commercial STPs from the US and Sweden, representing 80–90 % of the 2010 market share for all the major STP categories in these two countries, were analysed for the IARC Group 2A carcinogen acrylamide. These STPs comprised the following styles: Swedish loose and portion snus, US snus, chewing tobacco, moist snuff, dry snuff, soft pellet, hard pellet and plug.

**Results:**

Acrylamide was detected in all the products tested and quantified in all but one product. Concentrations ranged from 62 to 666 ng/g wet weight basis (WWB). The average levels of acrylamide (WWB) by type of STP were not significantly different (p > 0.05) except for US snus which had, on average, greater levels but with a very wide range of individual levels according to the manufacturer. Acrylamide levels in STPs were significantly and positively correlated with pH, but not with levels of either reducing sugars or ammonia nitrogen. Levels of acrylamide increased by sixfold or more (on a dry weight basis) during manufacture of a snus sample and then decreased during subsequent storage for up to 22 weeks. Acrylamide generation in tobacco generally appears to occur at lower temperatures, but longer time scales than found with food production.

**Conclusions:**

Acrylamide is a common contaminant of STPs, formed through heat treatment of tobacco. Our data show that exposure to acrylamide from consumption of STPs is small compared with exposure from food consumption or cigarette smoking.

**Electronic supplementary material:**

The online version of this article (doi:10.1186/s13065-015-0132-1) contains supplementary material, which is available to authorized users.

## Background

Smokeless tobacco products (STPs) have been collectively classified by IARC as carcinogenic to humans [1, 2]. However, there is evidence [3, 4] of substantial differences in the risk profiles of different STPs in approximate relation to their toxicant contents. There is emerging regulatory interest in establishing the comparative toxicant levels of different STPs. For example, the Food and Drug Administration (FDA) has assembled a list of 93 “hazardous or potentially hazardous constituents” (HPHC) of tobacco products, some of which have to be reported annually to FDA [5]. The list includes 79 designated as carcinogenic, as well as constituents that are toxic to the respiratory, cardiovascular and reproductive systems or addictive. One of the HPHC carcinogens on the list is acrylamide (CH_2_ = CHCONH_2_) which has been classified as a group 2A carcinogen (probably carcinogenic to humans) by IARC [6].

Acrylamide is a semi-volatile (vapour pressure of 0.007 mmHg at 25 °C and 0.07 mmHg at 50 °C [7]), water soluble chemical with a melting point of 84.5 °C. At temperatures above its melting point it polymerizes violently [8]. Acrylamide is used to manufacture polyacrylamide and grouting agents. It has been detected in tobacco smoke [9–12] and various cooked foodstuffs [13]. Generally acrylamide formation in foodstuffs occurs at temperatures >120 °C and concentrations increase with temperature and cooking times [13, 14]. It has also been found in a small number of tobacco and smokeless tobacco samples [15, 16]. Acrylamide uptake has been measured in human populations using the metabolites *N*-acetyl-*S*-(2-carbamoylethyl)-l-cysteine and *N*-(*R,S*)-acetyl-*S*-(2-carbamoyl-2-hydroxyethyl)-l-cysteine in urine [17]. The median levels of both metabolites were about two to four times higher in smokers than non-smokers, indicating that cigarette smoking is a significant source of acrylamide exposure.

Epidemiological studies of industrially exposed workers [18] found that acrylamide is a potent neurotoxin. However no evidence of significantly increased cancer rates in exposed workers has been found [6]. Studies in rats and other animals have found both neurotoxic [19] and carcinogenic effects of acrylamide [20].

We are currently conducting a comprehensive survey of toxicants in an extensive and varied set [21, 22] of contemporary STPs from the United States and Sweden. There is little information concerning the presence of acrylamide in smokeless tobacco products (STPs) other than levels in two Swedish snus products [15] and in two snus, two moist snuff and two dissolvable STPs in the US [16]. In order to understand the potential for STPs to act as a source of acrylamide exposure, we have measured the acrylamide content of 74 contemporary STPs commercially available in the United States and Sweden.

## Methods

### Tobacco samples

Tobacco samples were obtained in 2010. The products for analysis were chosen to reflect approximately 90 % market share of the major STP categories in the United States and Sweden at that time. In total, the survey comprised 31 Swedish products (10 loose snus and 21 portion snus) and 43 US products (13 chewing tobaccos, 5 dry snuffs, 2 hard pellet products, 1 soft pellet product, 15 moist snuffs, 6 US snus and 1 plug product). The Swedish products were sourced from Swedish retail websites and the US products were sourced from shops in the United States. After importation into the United Kingdom all samples were kept frozen at −20 °C until analysis.

#### Acrylamide

Acrylamide analyses were carried out by Labstat International (Kitchener, Ontario, Canada) using method TWT-336. The method used, which has been summarized previously [23], is an adaptation of the method used by Moldoveanu and Geraldi [16], from which more experimental details may be obtained. In summary, two grams of the ground and homogenized tobacco from a freshly opened package or tin was spiked with deuterated acrylamide internal standard (ISTD) and extracted with 20 mL water in a 50 mL flask on a mechanical shaker for 30 min. The supernatant was filtered through a 0.45 µm syringe filter into a 15 mL centrifuge tube collecting about 3 mL solution. To the tube was added 3–4 mL of dichloromethane for defatting and washing. The mixture was shaken by hand for 30 s and then centrifuged for 5 min. The aqueous solution was transferred to a test tube and 2 mL was purified by 2 stages of C-18 solid phase extraction (SPE).

Analytes were separated and detected using an AB Sciex (Framingham, MA, USA) API 3000 triple quad LC–MS/MS system with positive electrospray ionization (ESI) and operated in multiple reaction mode. A 5 µL aliquot of the sample was injected into the LC, and analytes separated using methanol and water as mobile phases. Three mass transition pairs (72/55, 72/54 and 72/44) were used for analyte confirmation and quantification. The most intense pair (72/55) was used for quantification, the two less intense transition pairs were used as qualifiers for further compound confirmation. STP acrylamide levels are reported in two ways, on an “as received” basis which we term “wet weight basis” (WWB), and also after correction for moisture content on a “dry weight basis” (DWB).

#### Moisture

Moistures of the STPs were determined at BAT using a gravimetric oven moisture method based on AOAC Method 966.02 [24] but using an oven temperature of 110 °C for 3 h rather than the 99.5 ± 0.5 °C specified by AOAC.

#### pH

1.0 ± 0.05 g of ground STP (1 mm meshed centrifugal grinding mill, but with cryomilling where necessary) was weighed into a round bottomed flask. A 50 mL aliquot of deionised water (greater than or equal to 18.2 MΏ resistivity) was added and the sample shaken for 30 min at 180 revs/min. The pH of the decanted extract was measured using a GL pH automated pH meter.

#### Reducing sugars

Tobacco reducing sugars were quantified using aqueous extraction of the STP sample, followed by continuous flow analysis. Reducing sugars were determined by the reduction reaction of reducing sugars with the cupric chelate of neocuproine in alkaline solution to form the highly coloured cuprous form, which is quantified spectroscopically at 460 nm.

#### Ammonia nitrogen

Ammonia nitrogen in tobacco was quantified spectroscopically using aqueous extraction followed by continuous flow analysis using a modification of the Bertholet reaction between ammonia, salicylate ions and dichloroisocyanurate, with nitroferricyanide as catalyst; the product, indophenol blue, was measured at 650 nm.

#### Snus production and aging study

Given the relatively high temperatures experienced by tobacco during snus manufacture (~100 °C for several hours [25]) it is of interest to understand how these tobacco heating steps impact acrylamide concentrations in a finished snus product. A controlled study was therefore conducted using a commercial BAT snus blend, processed in a pilot plant under normal BAT snus manufacturing conditions (consistent with manufacturing conditions reported previously [25]). The stability of acrylamide in snus under the environmental conditions experienced by commercial snus products (refrigeration at 4–8 °C) prior to sale was also examined.

In principle, each snus manufacturer may operate under proprietary manufacturing process conditions (e.g. presence of non-tobacco ingredients, processing temperatures and heating times), that incorporate the general heat treatment step [25], albeit with possible manufacturer-to-manufacturer variations in individual production steps. It is not possible to incorporate all possible manufacturing process variations into a single controlled study, therefore the findings of the current controlled snus processing and aging study must be regarded as indicative of events that can occur with snus rather than a precise reflection of events occurring with all snus products.

For the current snus processing and aging study, snus samples manufactured to BAT processing conditions were taken after the tobaccos and ingredients were blended but before heat treatment, and also sampled immediately after heat treatment. Processed snus was then packaged in sealed ziplock plastic bags and stored at 4–8 °C (reflecting storage conditions for snus in the retail supply chain in Sweden), prior to sampling after 8, 12, 16 and 22 weeks of storage. Samples were analysed for acrylamide and moisture content at all sampling times.

## Results

### Concentrations of acrylamide in STPs

Acrylamide concentrations in STP samples are shown in Table 1, both on a WWB and DWB. The moisture contents used to calculate the DWB concentrations of acrylamide are also shown in Table 1. Acrylamide was detected in all the samples analysed, and concentrations ranged from 62 to 666 ng/g WWB (82–756 ng/g DWB)—a tenfold range in the STPs examined. In only one product (Oomph Citrus Menthol P Snus) was the level too low to be quantified (>15 ng/g but <50 ng/g). The STP blend in the Oomph pouch was a lighter colour and contained a substantial content of a white material. Product packing informs as to the presence of cellulose powder and vegetable fibers amongst other ingredients, and a tobacco content which comprises 50 % of the total product mass. Hence the relatively low acrylamide content of this product may reflect the relatively low tobacco content.

**Table 1:** Product, manufacturer, moisture content, pH, reducing sugars, ammonia nitrogen and acrylamide concentrations in STPs

Product	Product type	Manufacturer	Moisture (%)	pH	Reducing sugars (%, WWB)	Ammonia nitrogen (%, WWB)	Acrylamide (ng/g)	
							WWB	DWB
*US STPs*								
Copenhagen Long Cut	MS	UST	52.3	8.1	<2.0	0.54	233	489
Copenhagen straight LC		UST	54.6	8.2	<2.0	0.38	212	466
Grizzly natural LC		CN	53.4	8.1	<2.0	0.29	192	411
Husky natural FC		UST	55.5	8.0	<2.0	0.56	135	303
Husky straight LC		UST	55.9	8.1	<2.0	0.37	131	297
Husky wintergreen		UST	55.1	7.5	<2.0	0.25	125	277
Kayak straight LC		SW	55.1	7.1	<2.0	0.11	85.7	191
Kodiak straight LC		CN	53	8.2	<2.0	0.21	155	329
Kodiak wintergreen		CN	52.5	8.4	<2.0	0.35	282	594
Silver creek		SW	54.9	7.0	<2.0	0.16	90.5	201
Skoal straight		UST	54.9	7.8	<2.0	0.24	141	312
Timberwolf natural FC		SM	51.3	8.0	<2.0	0.24	164	337
Timberwolf straight LC		SM	55.4	8.0	<2.0	0.26	218	488
Red seal natural FC		UST	54.4	7.8	<2.0	0.25	132	289
Red seal natural LC		UST	55	6.4	<2.0	0.57	187	415
*MS average*							161	349
Marlboro rich	US snus	PM	18.3	N/A	N/A	N/A	249	305
Marlboro peppermint		PM	12.6	N/A	N/A	N/A	562	643
Marlboro mild		PM	11.9	N/A	N/A	N/A	666	756
Marlboro spearmint		PM	13.8	N/A	N/A	N/A	595	690
Camel mellow		RJR	31.4	N/A	N/A	N/A	68.3	99.5
Camel frost		RJR	31.5	N/A	N/A	N/A	66.3	96.7
*US snus average*							368	432
Ariva Java (Pellet)	HP	SS	3.8	8.1	3.6	0	104	108
Stonewall wintergreen		SS	3.9	7.9	5.1	0	81.0	84.3
*HP average*							92	96
Oliver twist original	SP	HOT	20.4	5.3	2.1	0.25	83.8	105
Beech Nut	CT	SM	28.9	5.8	13.4	0.17	78.8	111
Chattanooga		SW	23.1	5.9	25.4	0.16	193	252
Durango		NA	25.5	6.0	13.4	0.17	106	142
Lancaster		SW	24.5	6.0	24.3	0.16	153	202
Levi garrett		CN	21.2	6.5	11.3	0.13	278	353
Morgans		CN	22.7	6.5	10.5	0.16	309	399
Redman gold		SM	24.3	6.1	8.5	0.15	77.2	102
Redman regular		SM	24.6	6.2	7.2	0.16	147	196
Southern pride		SM	25.1	6.1	9.8	0.17	160	213
Starr		SW	24.5	5.6	24.3	0.14	143	190
Stoker 707 Wintergreen		SM	24	5.8	13.3	0.13	62.2	81.9
Taylors pride		CN	22.1	6.3	8.0	0.16	157	201
Trophy		SM	25.9	6.1	8.4	0.16	164	221
*CT average*							156	205
Cannon ball	Plug	CN	20.4	5.3	12.0	0.12	122	153
Bruton	DS	UST	8.3	7.2	<2.0	0.38	131	143
Dental Sweet		CN	8.41	5.9	<2.0	0.21	90.4	98.7
Garrett		CN	7.98	6.0	<2.0	0.26	464	504
Honest		CN	9.07	6.2	<2.0	0.26	223	245
Square		SW	9.09	6.5	<2.0	0.41	124	136
*DS average*							192	213
*SWEDISH STPs*								
Catch Dry Euc. white mini	P snus	SM	21.4	8.6	1.1	0.12	185	241
General white		SM	53.4	8.7	0.6	0.1	161	346
General mini		SM	48.7	8.3	0.7	0.06	200	389
General		SM	48.3	8.5	0.9	0.07	199	385
Ettan		SM	49.6	8.5	0.3	0.04	276	547
Grov portion		SM	49.8	8.6	0.8	0.06	253	504
Grov white		SM	52.4	8.6	0.5	0.08	188	396
Goteborgs rape white		SM	52.9	8.5	0.8	0.08	172	365
Kronan		SM	46	8.2	0.8	0.05	193	357
Catch licorice mini		SM	49.3	8.5	1.1	0.17	155	305
Catch white licorice		SM	52.5	8.5	0.6	0.21	156	328
Granit white		F&L	43	7.5	0.6	0.20	74.0	130
Granit		F&L	48.8	8.5	0	0.11	104	203
Tre Ankare White		SM	53.3	8.7	0.1	0.09	131	280
LD portion original		JTI	48.8	8.7	0.1	0.06	201	392
Skruf strong		ITG	46.6	8.8	1	0.10	225	421
Knox portion		SM	46.3	8.0	1.4	0.10	109	202
Oomph citrus menthol		Northerner	64.7	N/A	N/A	N/A	NQ	NQ
T Romeo and Julieta Habanos		Habanos	52.9	8.3	0.1	0.11	165	351
1847 original		PMI	45	8.6	0.8	0.03	219	398
Gustavus Original		JTI	47.6	N/A	N/A	N/A	186	356
*P snus average*							190	368
General	L snus	SM	56.3	8.7	1.1	0.07	170	390
Ettan		SM	57.8	8.8	1.2	0.07	246	582
Grov		SM	55.9	8.7	0.9	0.06	183	414
Goteborgs rape		SM	56.6	8.4	1.2	0.06	172	396
Kronan		SM	57.3	8.8	1	0.05	167	391
Granit loose		F&L	54.6	8.3	0.5	0.19	202	446
LD		JTI	55.6	8.7	1.2	0.08	153	344
Skruf strong		ITG	54.3	8.1	0.7	0.10	203	445
Knox		SM	53.2	8.7	1.2	0.10	154	329
T Montecristo Habanos		Habanos	55.7	7.5	0.2	0.09	105	237
*L snus average*							175	397

Marlboro Rich, Peppermint, Mild, and Spearmint, Camel Mellow and Frost were not analysed for pH, reducing sugars and ammonia due to lack of sample availability

*NQ* Not quantified (Between LOQ and LOD)

Product Style: *MS* moist snuff, *HP* hard pellet, *SP* soft pellet, *CT* chewing tobacco, *DS* dry snuff, *LS* Loose Snus, *PS* Portion Snus

Manufacturers: *SM* Swedish match, *SS* Star Scientific Inc, *SW* Swisher, *PM* Philip Morris, *CN* Conwood, *UST* US Smokeless Tobacco Co, *HOT* House of Oliver Twist, *RJR* RJ Reynolds Co., *PMI* Philip Morris International, *Habanos*–Habanos Nordics, *F&L* Fiedler & Lundgren, *ITG* Imperial Tobacco Group, *JTI* Japan Tobacco International

**Fig. 1:**
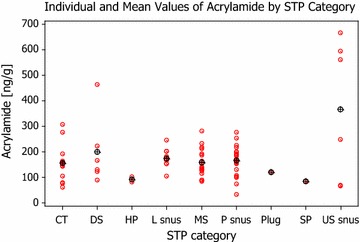
Mean and individual concentrations of acrylamide (ng/g WWB) by style of STP. Individual values are represented by *open red circles*, means by *black circles* with *crosses*

Average values of acrylamide by STP style are also shown in Table 1. US snus had the highest average acrylamide levels (368 ng/g WWB, 432 ng/g DWB) but there was a tenfold range of levels within this category (66–666 ng/g WWB). As a category the pellet products had the lowest acrylamide levels; the soft pellet product had a level of 84 ng/g WWB and the two hard pellet products had an average level of 92 ng/g WWB. The individual and average values by style of STP are shown in Fig. 1. An analysis of means indicated that the average WWB concentrations of acrylamide did not differ (p > 0.05) between STP styles except for US snus for which the higher average levels of acrylamide were significant. The significantly greater levels of acrylamide in the US snus category are due to the higher acrylamide levels found in the four products made by Philip Morris compared with the two RJ Reynolds products. The average DWB concentrations of acrylamide did not differ significantly (p > 0.05) between any of the STP styles except for a slightly lower average for the chewing tobaccos.

#### Composition of STPs and correlations with levels of acrylamide

The pH and levels of reducing sugar and ammonia nitrogen of the STPs are shown in Table 1. Limited sample availability prevented analysis of the 6 US snus and 2 of the portion Swedish snus samples. The correlation coefficients (r^2^) between the levels of acrylamide and pH, moisture content, reducing sugar and ammonia nitrogen for the 66 STPs analysed were calculated as follows:

*Moisture* There was no significant relationship (p > 0.05) between moisture and acrylamide concentration (r^2^ = 0.0042).

*pH* There was a significant (p < 0.05) and positive correlation between pH and levels of acrylamide measured on a DWB (r^2^ = 0.348). However on a WWB there was no significant correlation between pH and acrylamide concentrations (r^2^ = 0.036, p = 0.126).

*Reducing sugars and ammonia nitrogen* There was no significant correlation (r^2^ = 0.003) between ammonia nitrogen (%) and acrylamide (ng/g WWB) or between reducing sugars and acrylamide (r^2^ = 0.015).

**Fig. 2:**
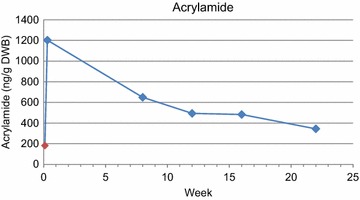
Effect of processing and storage on levels of acrylamide (DWB data) in snus. *Red diamond* pre-heating; *blue diamonds* post-heating

*STP production and aging study* The concentrations of acrylamide in the product pre- and post-heat treatment, and in the final product after storage for different times are shown in Table 2 and summarised (DWB data) in Fig. 2. Before heat treatment the snus blend had an average acrylamide level of 182 ± 9 ng/g DWB (167 ± 9 ng/g WWB). Following treatment, blend levels of acrylamide increased to 1202 ± 13 ng/g DWB (522 ± 6 ng/g WWB). Levels of acrylamide dropped to 344 ng/g DWB (150 ng/g WWB) after 22 weeks of storage at 4–8 °C. During the storage period the moisture content of the snus did not change, demonstrating that the storage container did not allow evaporative and diffusional losses from the snus samples over the 22 week period.

**Table 2:** Effects of processing and aging on acrylamide concentrations in snus

Sample	Oven moisture (%)	Acrylamide (ng/g WWB)		Acrylamide (ng/g DWB)	
		Average	St. dev.	Average	St. dev.
Snus mixer pre-treatment	8.39	167	9	182	9
Snus mixer post-treatment	56.5	522	6	1202	13
After 8 weeks storage	56.3	284	8	649	18
After 12 weeks storage	56.2	216	4	494	9
After 16 weeks storage	56.6	210	7	483	17
After 22 weeks storage	56.3	150	4	344	10

## Discussion

The results of this study demonstrate two clear findings: first, acrylamide is an ubiquitous contaminant of STPs, and second, its levels are not stable in tobacco, declining over time The latter finding is particularly notable, as the instability of acrylamide in tobacco has not been reported previously.

### Acrylamide stability in tobacco

Our experiments examining changes in acrylamide levels in snus samples during storage showed levels falling from 522 ng/g WWB immediately after manufacture to 150 ng/g WWB after storing for 22 weeks at 4–8 °C. These final levels were consistent with the levels measured from commercial samples in this study; these measurements suggest that the age of sample at point of analysis is an important influence on the levels measured, and that acrylamide contents of snus (and potentially other STPs) will change during its shelf-life. Further examination of the data shows that the loss of acrylamide was consistent with a first order loss process (r^2^ = 0.954), with a rate constant of 0.0551/day The half life of acrylamide in snus at 4–8 °C can therefore be estimated as about 12.5 days. The loss of acrylamide is unlikely to be due to evaporative loss, as the storage container did not allow the significantly more volatile species, water, to escape during the storage period. The losses of acrylamide are therefore likely to reflect reactions within the STP matrix during storage.

The stability of acrylamide in a variety of foodstuffs has been examined previously. For example Hoenicke and Gatermann [26] analysed 20 different foods for the effect of storage for 3 months on concentrations of acrylamide. Most of the foods including cookies, cornflakes, crispbread, raw sugar, potato crisps, instant coffee and peanuts were stable with respect to acrylamide levels after 3 months. However, significant decreases in acrylamide during storage were seen for ground coffee (dropping from 305 ± 21 µg/kg to 210 ± 13 µg/kg) and cacao (from 265 ± 25 to 180 ± 13 µg/kg). The authors discounted the possibility of evaporative losses and UV catalysed polymerisation since the coffee was stored in vacuum packages and it has been shown that even unpackaged food products show very low levels of acrylamide evaporation at temperatures less than 120 °C [27]. They concluded that reaction of acrylamide with thiol (–SH) and amine (–NH_2_) groups accounted for a large part of the acrylamide losses.

**Fig. 3:**
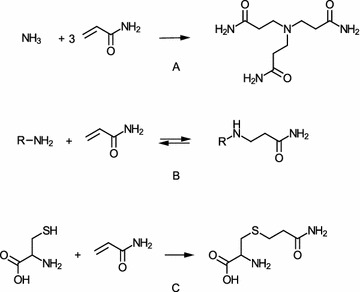
Potential reactions of acrylamide with tobacco components. **a** Reaction with ammonia to form 3,3′,3″-nitrilotrispropionamide. **b** Reaction amine groups to form 3-(alkylamino)propionamides. **c** Reaction with cysteine to form the addition product, cysteine-*S*-propionamide

Acrylamide undergoes thermally reversible reactions with amines, amino acids and polypeptides to form adducts via the Michael addition reaction. Primary and secondary amines yield the bis- and mono-adducts, respectively, while ammonia reacts with acrylamide to produce 3,3′,3″-nitrilotrispropionamide [28]. This latter reaction is shown in Fig. 3a. The reaction between acrylamide and amino acids to form 3-(alkylamino)propionamides is shown in Fig. 3b. Zamora et al. [29] suggested that reaction of acrylamide with amino acids may be the major mechanism by which acrylamide levels are reduced during storage of food products. The relatively high concentrations of ammonia, amino acids, proteins and amines [30] in tobacco make the above reactions with acrylamide possible during storage.

In tobacco, thiols, other than the sulphur-containing amino acid cysteine have not, to our knowledge, been reported [30]. The reaction of acrylamide with cysteine (Fig. 3c) to form the adduct, cysteine-*S*-propionamide, is used for characterising cysteine in protein sequence analysis [31]. Kobayashi et al. [32] have shown that cysteine and lysine as additives to foodstuffs are very effective for acrylamide removal at temperatures of less than 120 °C. However, free cysteine levels in tobacco are very low and it is therefore unlikely that thiols play a major role in acrylamide reduction during storage.

It is notable that reactions of acrylamide with ammonia and amines are likely to be sensitive to the pH of the STP, due to reduced chemical availability of these bases in more acidic STPs. Of the STPs examined in the present study MS, HP and snus samples had pH values predominately between 8 and 9, in contrast CT, SP, Plug and DS were predominately pH 5–6.5. It is therefore possible that the losses observed in this study for snus may not occur at the same rate with the more acidic STPs, and this is an area that merits further investigation.

Acrylamide losses of this kind may also have influenced the levels of acrylamide reported by Moldoveanu and Gerardi [16] and by Pérez and Osterman-Golkar [15]. It is therefore important that when analysing for acrylamide in STPs researchers take into account the age of the samples at the time of analysis.

### Processes leading to the formation of acrylamide

Given the ubiquity of acrylamide in the broad STP sample set of this study, it is important to understand how acrylamide is produced in tobacco. Factors contributing to its presence and levels in these STPs may well follow those established for food. It is therefore useful to briefly summarise understanding of this area.

Acrylamide in food or plant materials is generally viewed as being formed in Maillard reactions on heating to 120 °C and above for some minutes [13]. The levels of acrylamide in foods generally increase with increasing temperature and heating time up to temperatures of 160–180 °C. Prolonged heating at these temperatures, however, tends to decrease acrylamide levels [33]. It has also been shown that acrylamide can be formed at levels up to 100 ng/g at lower temperatures, even at ambient conditions, over long time periods in model systems, a variety of foods, animal feeds and environmental samples [27, 45].

**Fig. 4:**
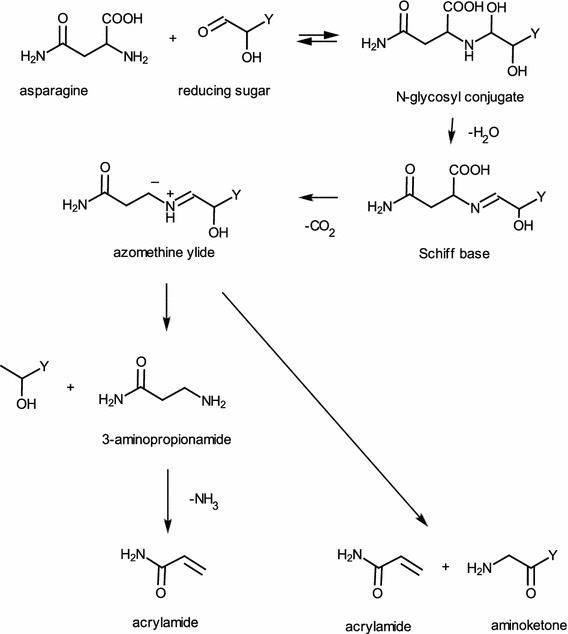
Formation of acrylamide from asparagine and reducing sugars (adapted from reference [36])

Major pathways for acrylamide formation in food involve the amino acid asparagine [34], either through direct decarboxylation and deamination [35], or through more efficient sugar mediated Maillard reaction routes [36] (Fig. 4). The *α*-amine group of asparagine reacts with the carbonyl of the sugar, forming a Schiff base that thermally decarboxylates to form an azomethine ylide, which thermally hydrolyzes to form 3-aminopropionamide; further degradation via elimination of ammonia forms acrylamide [36]. Asparagine can also be enzymatically decarboxylated to form 3-aminopropionamide without the involvement of reducing sugars [43]. Azomethine ylide can also decompose directly to form acrylamide and an imine [36, 37]. The importance of reducing sugars in acrylamide formation is supported by the high correlation between glucose and fructose levels in potatoes and the potential for acrylamide formation during frying [38–42]. In contrast there were no correlations found between asparagine levels and acrylamide in these studies, and it is generally thought that the concentration of reducing sugars is the limiting factor in the generation of acrylamide in food as long as asparagine is present.

Acrylamide may also be produced without the involvement of asparagine via the reaction of acrylic acid with ammonia derived from thermal decomposition of amino acids or ammonium salts. Although there is little data on its concentrations in foodstuffs, acrylic acid may be formed from acrolein or pyruvic acid. This is supported by observations [44, 45] that addition of ammonium carbonate (as a source of ammonia) to baked foods can lead to substantially increased acrylamide formation.

### Formation of acrylamide in tobacco

Learnings from studies of acrylamide formation in food processing suggests that understanding how acrylamide is formed in tobacco needs to focus on both how tobacco is heated, and on its chemical content.

### Heat treatment during tobacco processing and it’s relation to acrylamide content

Tobacco processing usually occurs at lower temperatures than those responsible for acrylamide generation during food preparation, although heating occurs over longer time-scales. In order to estimate the potential for these lower temperature tobacco heating processes to generate acrylamide we examined the data of Tareke et al. [46] for the rates of formation of acrylamide in potatoes at various temperatures (Additional file 1: Table S1). This is the most complete published data that we are aware of which allows for analysis of the kinetics of acrylamide formation in consumer products. Caution needs to be exercised in extrapolating reaction rates from one material (e.g. potato) to another (e.g. tobacco) as there are likely to be significant differences in the chemical contents (particularly acrylamide precursors) of the two different matrices. However, with this caution in mind, the data of Tareke et al. [46] is valuable in allowing a general assessment of the feasibility of low level acrylamide production in tobacco that experiences temperatures significantly lower than those involved in food production for significantly greater time periods.

We adjusted the acrylamide concentrations of Tareke et al. [46], to account for weight loss and background levels, together with heating times at different temperatures to calculate the Arrhenius parameters for this matrix. Regression analysis identified the 100 °C data point as an outlier, and therefore it was removed from this analysis. The remaining data points (120–220 °C) yielded a best fit regression line of ln (k) = −8862/T + 23.28, r^2^ = 0.98, where k (min^−1^) is the rate constant and T is the temperature (°K). This equation was used to estimate rate constants and acrylamide formation rates for potato at temperatures between 30 and 90 °C (Additional file 1: Table S2). The calculations suggest that at temperatures as low as 30 °C acrylamide levels at magnitudes consistent with those measured in tobacco (e.g. 100 ng/g) could be slowly generated over a period of a few weeks, and at 70–80 °C these acrylamide levels could be generated over a period of several hours.

There is limited information available in the literature concerning levels of acrylamide in tobacco and tobacco products (shown on a WWB in Table 3). Pérez and Osterman-Golkar [15] measured acrylamide in 2 brands of Swedish portion snus, while Moldoveanu and Gerardi [16] measured acrylamide in 12 samples of tobacco, including uncured leaf, cured tobacco leaf, cigarette tobacco blends, a dissolvable STP blend, a US snus blend and several finished STPs. The samples analysed by these authors and ourselves collectively cover a wide variety of tobacco and tobacco product types, and we have used the combined data set to understand the sources of acrylamide in different tobacco products.

**Table 3:** Literature values of acrylamide found in tobacco products

	Acrylamide (ng/g WWB)		
	Value or range (no. of samples)		
	Reference [15]	Reference [16]	Present study
Swedish snus	710–2414 (2)		NQ-276 (31)
US snus		70–83 (2)	66–666 (6)
Moist Snuff		87–180 (2)	86–282 (16)
Camel strips fresh		126 (1)	
Camel sticks mellow		367 (1)	
Camel sticks mellow blend pre-manufacture		130 (1)	
Flue-cured leaf		50–52 (2)	
Burley leaf		87 (1)	
Mixed stem		46 (1)	
Uncured dried tobacco		N/D (1)	
HP			81–104 (2)
SP			84 (1)
DS			90–223 (5)
CT			62–309 (13)

*N/D* not detected, *HP* hard pellet, *SP* soft pellet, *DS* dry snuff, *CT* chewing tobacco

To aid understanding and interpretation of these data we have adapted the five-step generalised model for STP production described by Wahlberg and Ringberger [47]; our revised model accommodates a wider time-frame in the production and sale of STPs, as follows:

- tobacco cultivation,
- curing and selection of cured tobaccos
- post-curing tobacco processing, leaf shredding and blending
- STP manufacture
- storage, distribution and sale.

A generalised version of this model (other than step d) relates equally to the production of cigarette tobaccos, and therefore data for these tobaccos were included in the following discussion.

- Tobacco cultivation,
  It is well established [48, 49] that uncooked and unheated plant materials contain no measureable levels of acrylamide (e.g. raw potatoes, dried fruit, oatflakes and wheat flour were reported by Lingnert et al. [48] to be <30 ng/g, while Delgardo-Andrade et al. [49] reported acrylamide levels of a number of fruits to be <3 ng/g). Consistent with this, Moldoveanu and Gerardi [16], using an analytical method with a limit of detection of 12 ng/g, failed to detect acrylamide in uncured dried tobacco.
- Curing and selection of cured tobaccos
  STPs may contain air cured, fire cured and/or flue cured tobaccos. For air cured and fire cured tobaccos, the curing process—wilting, yellowing and drying—takes approximately 6–8 weeks, during which the tobacco is subjected to temperatures typically 30 ± 3 °C with temperatures not allowed to exceed 38 °C [50]. The fire curing process additionally involves subjecting the tobacco to wood smoke after the yellowing stage [51]. Flue-curing is a much shorter process. Yellowing is typically carried out at 35 °C for 2 days then the temperature is raised over the next 6 days to a maximum of 72 °C for the drying stage [52]. Our analysis of the data reported by Tareke et al. [46] suggests that these conditions are conducive to the generation of low levels of acrylamide during both these time-scales. Moldoveanu and Gerardi [16] found that samples of flue-cured, air-cured and mixed stem tobaccos contained between 46 and 87 ng/g of acrylamide, a level substantially higher than measured in uncured tobacco.
- Post-curing tobacco processing, leaf shredding and blending
  Post-curing, tobaccos intended for cigarette production are often stored at ambient temperatures for extended periods of time. Prior to storage the leaf is processed in a “threshing process” wherein tobacco leaf is heated to 45–60 °C prior to the separation of soft leaf material from the leaf mid-rib or “stem” [53]. Post-storage, and during cigarette production, the tobaccos are generally moistened, blended, cut to target particle size and dried to a manufacturing moisture prior to assembly into cigarettes [54]. During this latter processing operation tobacco temperatures can reach 70–80 °C for some minutes. The acrylamide measurements of Moldoveanu and Gerardi [16], of 50–120 ng/g for five cigarette tobacco blends, suggest that these processing stages have a small contribution to acrylamide contents of cigarettes.
  It is likely that the tobaccos used in STP manufacture also follow these general processes. It is challenging to directly examine the impact of these steps on STP tobaccos from a product survey of the kind reported in this work as different tobacco product manufacturers operate with proprietary manufacturing practises. Therefore to better understand the influence of post-curing tobacco processing on acrylamide levels, we also analysed the acrylamide content of blended tobaccos sampled from a snus manufacturing line immediately prior to heat treatment. Before heat treatment the snus blend sample analysed in this work had an average acrylamide level of 167 ± 9 ng/g WWB. Similarly, the concentration of acrylamide in the tobacco used to make Camel Sticks Mellow was reported by Moldoveanu and Gerardi [16] to be 130 ng/g, These values are higher than the range of levels reported for cured tobaccos and further supports the likelihood of acrylamide generation in the tobacco processing steps pre-STP manufacture.
- STP manufacture

#### Snus

Comparing our results with those for similar products reported in the two earlier studies, acrylamide levels found in Swedish snus in our study are considerably lower (NQ-276 ng/g WWB) than those found in 2003 by Pérez and Osterman-Golkar. Our results for two US snus brands manufactured by RJR (66–68 ng/g WWB) are comparable to those found by Moldoveanu and Gerardi, while our results for four brands manufactured by Philip Morris are considerably higher (249–666 ng/g WWB). The wide range of levels measured in snus samples, both historically and when comparing samples manufactured by different producers, may reflect an important influence of snus production methods on the generation of acrylamide. This view is supported by experiments described in a patent by RJ Reynolds [55] concerning the use of additives to inhibit acrylamide formation during heat processing of snus-like STPs.

The snus process involves grinding tobacco, mixing it with water and salt, and heating the mixture at 80–100 °C for several hours before cooling [25]. Then ingredients such as flavours, humectants and sodium carbonate are added and, in Sweden, the finished product is packed and stored at 4–8 °C. Clearly, the elevated and sustained temperatures involved in snus manufacture may contribute to acrylamide formation. To evaluate the contribution of sustained high temperatures during processing to acrylamide formation we examined the concentration of acrylamide in tobacco before and after snus manufacturing (Table 2; Fig. 2). As noted above, before treatment the snus blend had an average acrylamide level of 167 ng/g WWB. Following treatment, blend levels of acrylamide increased to 522 ng/g WWB demonstrating a major influence of heating the snus mixture to these elevated temperatures. Significant changes in moisture content occur in the snus production process, and expressing the data on a dry-weight basis to allow for these changes showed more significant increases from 182 ng/g to 1202 ng/g DWB. Clearly, the extended heating involved in snus production can generate high levels of acrylamide in tobacco.

#### Moist snuff

Moldoveanu and Gerardi [16] also reported that one moist snuff brand had 180 ng/g acrylamide, which is within the range of moist snuff results (86–282 ng/g) measured in this study. Moist snuff features a blend of fire-cured and dark air-cured tobaccos, and is manufactured through a fermentation process in closed vessels during a period of several weeks, with pH and temperature monitoring [47]. As noted above, when tobacco is exposed to moderately elevated temperatures for extended periods of time acrylamide can be generated. The threefold range of values observed in the moist snuff samples (Table 1) may well reflect differences in tobacco production practices between different manufacturers, as well as possible sample age at the time of analysis. However a more extended and controlled study would be required to establish the robustness of these observations.

#### Dry snuff

There is relatively little detailed information available concerning the preparation of modern US dry snuff products, other than descriptions referring to the use of fermented fire-cured tobaccos [56]. However, IARC Monograph 89 [1] describes fermentation periods lasting 2 months for dry snuff products manufactured in the mid twentieth century. The levels of acrylamide measured in this work cover a wide range of values (90–464 ng/g), with both highest and lowest values observed from the same manufacturer.

#### Chewing tobaccos and plug

The acrylamide levels in the chewing tobaccos measured in this study covered a range of 62–309 ng/g WWB, with some suggestion of different levels between manufacturers and/or products. Loose leaf chewing tobaccos are subjected to “sweating” at slightly elevated temperatures for an extended period of time [47], and it is anticipated that this process, which may differ between manufacturers, could promote acrylamide formation. Plug chewing tobacco is not reported to be subjected to this sweating stage, and the acrylamide level we measured in the plug sample was lower than many of the loose leaf chewing tobacco samples.

#### Dissolvable STPs

Moldoveanu and Gerardi [16] reported that Camel Strips Fresh and Camel Sticks Mellow had 126 and 367 ng/g acrylamide respectively. As noted above, the concentration of acrylamide in the tobacco used to make Camel Sticks Mellow was approximately one-third that of the level in the finished STP, indicating that acrylamide is formed during the manufacture of this product. The manufacturing process for Camel Sticks has been reported [57] to feature an extrusion stage, which generally features elevated temperatures for a short period of time, and may therefore have contributed to the level of observed acrylamide. The two dissolvable (hard pellet) products measured in this study were associated with relatively low levels of acrylamide (81–104 ng/g).

Taken together, the acrylamide levels measured in STPs suggest that different manufacturing processes, particularly the steps featuring elevated temperatures, may have a strong influence on acrylamide levels in STPs.

#### Storage, distribution and sale

Our ageing study demonstrates the instability of acrylamide in a snus tobacco matrix, with slow losses in acrylamide levels over time. The losses in other STP matrices have not been investigated, but similar behaviours are likely given the commonality of the species that acrylamide reacts with across tobacco types and products. However, the extent of acrylamide losses may differ significantly in magnitude because of the pH differences noted above, and also the differences in environmental conditions between manufacture and storage. For example, Swedish snus is stored between 4 and 8 °C prior to sale [25] whereas US STPs are exposed to a range of ambient conditions and durations. Clearly, this represents a complex backdrop against which to understand sample ageing and acrylamide losses, and is an area meriting further investigation.

### Tobacco chemistry and its relationship to acrylamide levels

Given the emphasis above on the slow low-temperature development of acrylamide in tobacco, it is necessary to understand why uncured tobacco contains undetectable levels of acrylamide. The answer to this question lies with the large scale changes that occur in tobacco leaf chemistry as the tobacco cures. During senescence and curing, levels of asparagine, which is the major nitrogen transport and storage amino acid in tobacco, increase rapidly [58] as proteins are broken down. It is thought that ammonia released during protein hydrolysis also results in the production of asparagine and glutamine. As noted above, asparagine has been identified in food studies as the major precursor of acrylamide, and lower levels of asparagine in tobacco during plant growth will result in lower rates of acrylamide generation.

In contrast, substantial increases in asparagine levels in tobacco during curing can increase the potential for acrylamide generation. Support for the occurrence of Amadori and Maillard-style reactions during low temperature curing comes from the isolation of various sugar- amino acid compounds from cured tobacco, including 1-deoxy-l-asparagino-fructose [59].

The importance of asparagine as an acrylamide precursor during snus manufacture is also indicated by a patent by RJ Reynolds [55], which describes the use of additives to inhibit acrylamide formation during heat processing of snus-like STPs. The additives, which include asparaginase as well as amino acids and compounds with thiol groups, are added to the STP formulation prior to heat processing and have been shown to significantly reduce acrylamide concentrations. Asparaginase, for example, which converts asparagine to aspartic acid, was reported to reduce the acrylamide level in the processed STP by 67 %, when added to the formulation at 250 ppm. The patent also shows that lowering the pH of the formulation from 8.7 to 6.5 by removing sodium hydroxide, was reported to cause a 93 % reduction in the acrylamide level. These patent data are consistent with our findings that there is a correlation between pH and acrylamide levels as found for foods.

Analysis of our samples did not show any correlation between levels of reducing sugars and acrylamide. This contrasts with the strong correlations between reducing sugars and subsequent formation of acrylamide in potatoes. This may point to the importance in tobacco of the enzymatic decarboxylation of asparagine as a source of acrylamide. However, it should also be noted that the sugar levels determined in the present study, may not provide full insight into the operation of the sugar/asparagine mechanism due to differences in sample age. Another source of acrylamide, acrylic acid, has not been identified in tobacco [30], and therefore cannot be viewed at this time as a major source of acrylamide in tobacco products.

### Acrylamide exposure from food and STPs

Acrylamide contents have been reported across a wide range of food materials [13], with particularly high levels of acrylamide reported in coffee, cooked potato and bakery products. In an 8 country dietary survey [60] these food types generally contributed to around 90 % of the total mean dietary exposure. National and regional studies have reported national-level mean adult daily exposures of 0.2 to 1.0 µg/kg bw, which has led to estimates of mean daily dietary acrylamide exposures of 1 μg/kg of body weight (bw)/day for general populations (including children), and 4 μg/kg of bw/day for consumers with a high dietary exposure [60].

Using the mean acrylamide content for Swedish snus of 170 ng/g, combined with a daily consumption of 14 g for pouched snus and 32 g for loose snus [61], and an estimate for the amount extracted during use of 33 % [62] gives estimates of daily per capita intake of 0.8 and 1.8 μg/day for pouched and loose snus respectively. Using published body mass data by country [63] intake per unit body weight ranges from 9 ng/kg bw/day for a US male using pouched snus to 27 ng/kg bw/day for a Swedish female using loose snus. Assuming similar consumption levels, American moist snuff and chewing tobacco products likely result in similar exposures. Acrylamide exposure from other types of STP product will depend both on their acrylamide contents, and also their usage patterns for which there is a shortage of published quantitative data.

Exposure through use of contemporary smokeless tobacco products is therefore likely to be small in comparison to dietary exposure, in contrast to the significant exposure to acrylamide from cigarette smoking [17]. Studies have reported 1.7 to 4 times the level of acrylamide biomarkers in the urine and blood of smokers compared to non-smokers [17, 64], and there have been daily exposure extimates of 3 µg/kg bw acrylamide uptake from cigarette smoking [65].

## Conclusions

In this study we determined acrylamide levels in 74 samples of STP from Sweden and the US. Our survey showed that acrylamide was present in all analysed samples, with a greater than tenfold range in acrylamide contents amongst the measured STPs. There were no significant differences between the average levels for different STP categories, except for US snus. The latter category contained individual brands with the lowest and highest levels of acrylamide observed in this study and products with similar acrylamide levels appeared to be grouped by manufacturer Acrylamide levels in Swedish-style snus were found to decline with sample age post-manufacture, consistent with chemical reactions within the STP matrix, and therefore exposure of consumers to acrylamide during snus use is likely to be greatest with freshly manufactured products.

Examination of mechanistic factors underlying acrylamide production in tobacco showed behaviour consistent with slow generation of acrylamide at lower temperatures, but longer time scales, than encountered during food production. Consideration of tobacco heat treatments during post-harvest processing steps identified a number of events where acrylamide may be generated in the manufacture of tobacco products; lowest levels were found in cured tobacco prior to processing, and highest levels immediately post snus manufacture. The acrylamide levels of different tobacco products were consistent with the role of asparagine as the direct pre-cursor of tobacco acrylamide, but no correlation was found with reducing sugar levels. Calculations demonstrated that STPs are a minor source of acrylamide exposure compared with diet or cigarette smoking.

## Additional file

10.1186/s13065-015-0132-1 Kinetic analyses of acrylamide formation in potatoes. **Table S1.** Data on acrylamide formation in potatoes. **Table S2.** Calculated rate constants for acrylamide formation in potatoes at 30–90 °C.

## Abbreviations

BLD: below the limit of detection
DWB: dry weight basis
FDA: US Food and Drug Administration
HPHC: harmful and potentially harmful constituents
IARC: International Agency for Research in Cancer
LOD: limit of detection
LOQ: limit of quantification
RSD: relative standard deviation
STP: smokeless tobacco product
WWB: wet weight basis
