# Supplementary material for: The acrylamide content of smokeless tobacco products
Source: Chem Cent J. 2015 Oct 12;9:56. doi: 10.1186/s13065-015-0132-1 (PMC4602115; doi:10.1186/s13065-015-0132-1)
Supplement: Supplementary file 1 — 10.1186/s13065-015-0132-1 Kinetic analyses of acrylamide formation in potatoes. Table S1. Data on acrylamide formation in potatoes. Table S2. Calculated rate constants for acrylamide formation in potatoes at 30–90 °C. [file 13065_2015_132_MOESM1_ESM.docx]

Supplementary Materials:

Supplementary Table 1. Data on acrylamide formation in potatoes from reference 46.

| Max T  (°C) | 1/T  (°K^−1^) | Acrylamide (µg/kg) | Heating time (min) | Weight loss (%) | Acrylamide corrected (µg/kg) | Rate (µg/kg/min) | ln Reaction rate |
| --- | --- | --- | --- | --- | --- | --- | --- |
| control |  | 146 | 0 | 0 | 146 |  |  |
| 100 | 0.00268 | 173 | 19 | 14.9 | 147 | 0.064 | -2.74 |
| 120 | 0.00255 | 217 | 18.2 | 19.9 | 174 | 1.53 | 0.42 |
| 140 | 0.00242 | 376 | 17.4 | 29.8 | 264 | 6.80 | 1.91 |
| 160 | 0.00231 | 808 | 16.6 | 41.9 | 470 | 19.5 | 2.97 |
| 180 | 0.00221 | 1965 | 15.8 | 49 | 1002 | 54.2 | 3.99 |
| 200 | 0.00211 | 3479 | 15.0 | 54.3 | 1591 | 96.3 | 4.57 |
| 220 | 0.00203 | 5051 | 14.2 | 55 | 2273 | 149.8 | 5.01 |

Supplementary Table 2. Rate constants calculated at 30-90°C from data in reference 46.

| Max T  (°C) | Reaction Rate (µg/kg/min) | 1/Reaction Rate (min/µg/kg) | Time (days) to generate 100 ng/g acrylamide |
| --- | --- | --- | --- |
| 90 | 0.308 | 3.25 | 0.23 |
| 80 | 0.153 | 6.52 | 0.45 |
| 70 | 0.0733 | 13.6 | 0.94 |
| 60 | 0.0335 | 29.8 | 2.1 |
| 50 | 0.0146 | 68.4 | 4.8 |
| 40 | 0.0060 | 166 | 11.5 |
| 30 | 0.0024 | 425 | 29.5 |
